# Weak Defect Identification for Centrifugal Compressor Blade Crack Based on Pressure Sensors and Genetic Algorithm

**DOI:** 10.3390/s18041264

**Published:** 2018-04-19

**Authors:** Hongkun Li, Changbo He, Reza Malekian, Zhixiong Li

**Affiliations:** 1 School of Mechanical Engineering, Dalian University of Technology, Dalian 116024, China; lihk@dlut.edu.cn; 2 Laboratoire Vibrations Acoustique, University of Lyon, INSA-Lyon, LVA EA677, Villeurbanne F-69621, France; 3 Department of Electrical, Electronic & Computer Engineering, University of Pretoria, Pretoria 0002, South Africa; 4 School of Mechanical, Materials, Mechatronic and Biomedical Engineering, University of Wollongong, Wollongong, NSW 2522, Australia; zhixiong.li@ieee.org

**Keywords:** centrifugal compressor, pressure sensor, defect identification, continuous wavelet transform, stochastic resonance, woods-saxon and Gaussian, genetic algorithm

## Abstract

The Centrifugal compressor is a piece of key equipment for petrochemical factories. As the core component of a compressor, the blades suffer periodic vibration and flow induced excitation mechanism, which will lead to the occurrence of crack defect. Moreover, the induced blade defect usually has a serious impact on the normal operation of compressors and the safety of operators. Therefore, an effective blade crack identification method is particularly important for the reliable operation of compressors. Conventional non-destructive testing and evaluation (NDT&E) methods can detect the blade defect effectively, however, the compressors should shut down during the testing process which is time-consuming and costly. In addition, it can be known these methods are not suitable for the long-term on-line condition monitoring and cannot identify the blade defect in time. Therefore, the effective on-line condition monitoring and weak defect identification method should be further studied and proposed. Considering the blade vibration information is difficult to measure directly, pressure sensors mounted on the casing are used to sample airflow pressure pulsation signal on-line near the rotating impeller for the purpose of monitoring the blade condition indirectly in this paper. A big problem is that the blade abnormal vibration amplitude induced by the crack is always small and this feature information will be much weaker in the pressure signal. Therefore, it is usually difficult to identify blade defect characteristic frequency embedded in pressure pulsation signal by general signal processing methods due to the weakness of the feature information and the interference of strong noise. In this paper, continuous wavelet transform (CWT) is used to pre-process the sampled signal first. Then, the method of bistable stochastic resonance (SR) based on Woods-Saxon and Gaussian (WSG) potential is applied to enhance the weak characteristic frequency contained in the pressure pulsation signal. Genetic algorithm (GA) is used to obtain optimal parameters for this SR system to improve its feature enhancement performance. The analysis result of experimental signal shows the validity of the proposed method for the enhancement and identification of weak defect characteristic. In the end, strain test is carried out to further verify the accuracy and reliability of the analysis result obtained by pressure pulsation signal.

## 1. Introduction

During the operation of a centrifugal compressor, the blades suffer from combined effects of centrifugal force, unsteady flow and vibration and so forth [1]. Therefore, as the most sensitive part in a compressor, blades are exposed to periodic vibration and fluid excitation for the long time [2]. At the same time, with the development of turbo-machinery, the working environment for the impeller is more and more complex. Consequently, the blades are more susceptible to cracks and other defects. The defect information of blades is usually difficult to identify at an early stage of the crack but the blade may fracture at any time in the late stage which will lose meaning for condition monitoring. Therefore, how to detect the crack defect of blades dynamically with a relatively simple and high accuracy method is one of the complex problems in fault diagnosis field. Generally, displacement sensors are used to detect the shaft defect [3,4] but the blade crack is always difficult to identify by shaft vibration signal because of the complex transfer path. Compared to the shaft crack, the identification for blade crack is more difficult. 

Many scholars studied the blade condition monitoring with different ways. For example, Rao et al., proposed a method to extract the characteristic information from vibration signal of the gas turbine for recognizing the blade state [5]. Witek studied the development process of the blade crack by using a vibration signal obtained in the laboratory [2]. Egusquiza et al. studied the blade failure mechanism of a turbine pump, which provides the theoretical foundation for blade characteristic extraction [6]. In addition, acoustic emission signal is also investigated for the defect classification of turbine blades [7]. Support vector machine has been proved to have good performance on shaft crack classification and prediction, therefore, it was tried to diagnose the blade damage of a helicopter [8]. At the same time, König conducted a series of studies on the rotating impeller failure mechanism through a pressure pulsation signal, which provided good results and confirmed that the vibration information of the blade can be reflected in the pressure pulsation signal [9,10]. However, most of the research is focused on the gas turbine currently, although there exists a certain similarity between centrifugal compressors and gas turbines. Moreover, most of the research hasn’t focused on the blade on-line condition monitoring, especially by using pressure pulsation signal. Considering the interaction between the blade and the airflow nearby during the operation of the compressor, pressure pulsation signal close to the rotating impeller may well reflect the health states of the blade and therefore is selected for the identification of blade crack in this paper.

A big problem should be noted is that the characteristic information representing the blade crack is often submerged in the complex background noise, which will lead to the weak defect characteristic difficult to identify from pressure pulsation signal. Therefore, it is necessary to study the method that can effectively extract the weak crack information from the test signal with strong background noise. Restraining noise seems to be a common and effective method, which can inhibit the noise and increase the signal-to-noise ratio (SNR). However, it is not useful for the weak signal with strong noise. Stochastic resonance (SR) can provide a new idea compared to the traditional noise suppression methods. That is because the stochastic resonance is able to transfer part of noise energy to the weak characteristic through a nonlinear system. As a result, it can improve the quality of weak signal [11]. So, the stochastic resonance method utilizing noise reasonably can be considered for the weak defect characteristic frequency enhancement of the cracked blade. Stochastic resonance was first put forward by Benzi and his partners in 1981, and was used to explain the glacial periodically recurrent phenomenon in the Earth’s meteorology occurring every 100 thousand years [12]. Subsequently, many scholars found that the stochastic resonance is also conducive to extract weak characteristics from strong noise and then this nonlinear phenomenon has been widely used in the identification of early weak fault [13,14,15,16,17,18]. Wherein, He et al., studied the multi-scale noise tuning methods [19]. Qiao et al. considered the influence of potential asymmetries on stochastic resonance that was subject to both multiplicative and additive noise [20]. Qin et al., found the frequency range selection characteristic of re-scaling frequency stochastic resonance because of the driving frequency limitation of bistable stochastic resonance and then separated vibration components with different frequencies by iteratively using stochastic resonance [21]. Hu and Li applied an adaptive stochastic resonance method to diagnose crack defect [22]. However, there is seldom reference in the literature about the centrifugal compressor blade crack detection by using stochastic resonance methods.

This paper uses CWT to filter the sampled signal around blade passing frequency first and then utilizes Gaussian potential (GP) to change Woods-Saxon single stable stochastic resonance system into a bistable stochastic resonance system for extracting the weak characteristic frequency excited by blade crack defect. GA is used to optimize the related parameters. The method is validated with the analysis of simulation signal and actual test signal. The detailed structure is shown as follows: Section 2 introduces the theory of continuous wavelet transform. Section 3 describes the basic principles of the potential well model and the theory of bistable stochastic resonance system based on the combined Woods-Saxon and Gaussian potential (WSG). Section 4 presents the simulation signal with strong noise and characteristic frequency analysis by CWT-envelope method and CWT-WSG stochastic resonance method respectively. Section 5 introduces the application of the proposed method on blade crack detection of the centrifugal compressor with pressure pulsation signal. Strain test is further carried out to verify the correctness of the result. The conclusions are given in Section 6.

## 2. Continuous Wavelet Transform

Wavelet transform developed from the traditional Fourier transform is a local transform in time and frequency domain which can extract the details of signal through multi-scale analysis. It has been intensively investigated and applied to extract fault characteristics of mechanical equipment from its vibration signal as it can effectively filter noise and preserve characteristics information. In this paper, continuous wavelet transform is used to pre-process the obtained experimental signal and its definition is shown as Equation (3). Set ψ(t) as a finite energy function $\psi \left(t\right)\in L^{2}(R)$, if its Fourier transform $\hat{\psi }(\omega )$ could satisfy the conditions of permissibility shown by Equation (1)

(1) $$C_{\psi }=\int {\left|\hat{\psi }(\omega )\right|}^{2}/\left|\omega \right|d\omega <\infty$$

Then ψ(t) is referred to as a mother wavelet. Expand and translate the mother wavelet ψ(t) and set a to be the scale factor and b to be the translation factor. Then, $\psi_{a,b}\left(t\right)$ shown by Equation (2) is the function after stretched and translated.

(2) $$\psi_{a,b}\left(t\right)={\left|a\right|}^{-1/2}\psi \left(\frac{t-b}{a}\right)$$

$\psi_{a,b}\left(t\right)$ above depending on the stretching parameters and translation parameters is called the wavelet function. And CWT of the continuous time signal $x(t)\in L^{2}\left(R\right)$ is defined as the inner product of the signal and wavelet function.

(3) $$W_{x}\left(a,b;\psi \right)=a^{-1/2}\int x(t)\psi^{*}\left(\frac{t-b}{a}\right)dt$$

Wherein $\psi^{*}(t)$ is the conjugate of ψ(t). According to Equation (3), it can be found the signal will be projected onto the two-dimensional time-scale plane after the CWT. The wavelet transform coefficients actually reflect the similarity between the local signal and the wavelet function, which means the larger the coefficient is, the greater similarity they have. In addition, the continuous wavelet inverse transform is shown as follows, which can be used to reconstruct the processed signal.

(4) $$x(t)=\frac{1}{C_{\psi }}\int \int a^{-2}W_{x}(a,b;\psi )\psi_{a,b}dadb$$

The inverse transform equation indicates that there is no energy loss for CWT and the energy is conserved. Thus, Equation (5) is valid. Moreover, the modulus of the signal’s CWT coefficient SG(a,b;ψ) is defined as Equation (6) which can usually be used for further analysis. As CWT is a multi-scale analysis method, different scales correspond to different frequencies. In this paper, CWT is used for pressure pulsation signal to obtain the wavelet scale spectrum first, then the scales around blade passing frequency are selected to reconstruct time domain signal for the purpose of filtering noise and preserving the characteristic information induced by blade crack defect.

(5) $$\int {\left|x(t)\right|}^{2}dt=\frac{1}{C_{\psi }}\int a^{-2}da\int {\left|W_{x}\left(a,b;\psi \right)\right|}^{2}db$$

(6) $$SG(a,b;\psi )={\left|W_{x}(a,b;\psi )\right|}^{2}$$

## 3. Stochastic Resonance

Woods-Saxon potential (WSP) and GP are combined to form the new potential function of bistable stochastic resonance system which will be described in Section 3.4. The WSP is a monostable potential with three parameters—potential height, width and steep degree controlling the shape of WSP. Moreover, for the same input, different potentials always produce different outputs. GP satisfies symmetry, continuity and boundness, so it is chosen as the potential barrier and changes the Woods-Saxon monostable potential function into a bistable potential function.

### 3.1. Woods-Saxon Potential Well Model

The potential function $U_{ws}(x)$ in the WSP well model is a nonlinear symmetric potential proposed by Woods and Saxon. It is also used by Deza and others for the collection of energy at first. The model can be expressed as follows:

(7) $$U_{\mathrm{WS}}(x)=-\frac{V_{1}}{1+\exp((\left|x\right|-R_{1})/a)}$$

**Figure 1:**
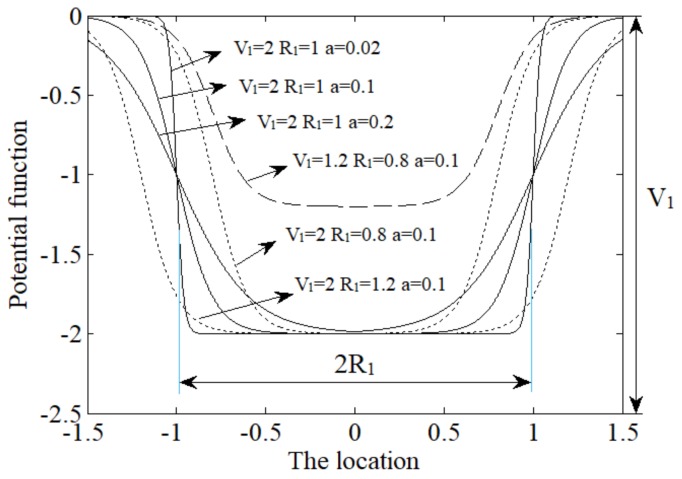
The shape of the potential function with different parameters.

Wherein, $V_{1}$ is the well depth, $R_{1}$ is the well width and a is the steep degree of the potential. These three parameters determine the shape of the potential which also means they can determine the energy that the oscillator can obtain. Figure 1 shows the effect of different parameters on the shape of the potential function. The three solid lines in the figure correspond to the change of parameter a (parameter $V_{1}$ and parameter $R_{1}$ are fixed), the influence of parameter a on the potential function can be seen from the solid lines. When a is smaller the potential will be steeper. Two point lines correspond to fixed $V_{1}$, a and varying $R_{1}$, the influence of parameter $R_{1}$ on the well width can be seen by comparing these lines. There also exists a dotted line corresponding to the varying $V_{1}$, it shows the effect of $V_{1}$ on the well depth. Therefore, unlike the potential in classical bistable stochastic resonance system, the parameters of WSP change the shape of the potential independently. They are not coupled together. 

### 3.2. The Potential Well Model of Gaussian Potential (GP)

The radial GP well model can be expressed as follows:

(8) $$U_{G}(x)=-V_{2}\exp\left(-\frac{x^{2}}{R_{2}^{2}}\right)$$

**Figure 2:**
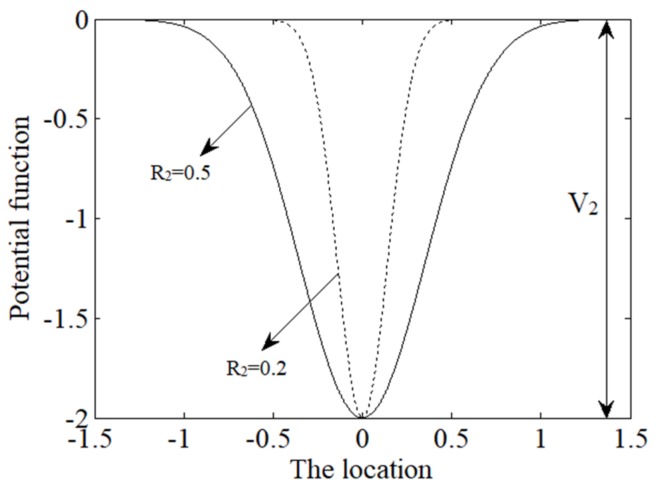
The shape of Gaussian potential function with different parameters.

Wherein, $V_{2}$ represents the depth of the potential well and $R_{2}$ represents the width. Figure 2 shows the influence of different parameters on the potential function. It can be seen from the figure that the potential well will be steeper when $R_{2}$ is smaller. 

### 3.3. The Combined Potential Model

The WSP and GP models mentioned above can be combined into a new bistable potential well model. And the new WSG potential function can be expressed as follows:

(9) $$\begin{array}{cc} U(x)=U_{ws}(x)-U_{G}(x)= & -\frac{V_{1}}{1+\exp((\left|x\right|-R_{1})/a)}\\ & +V_{2}\exp(-\frac{x^{2}}{R_{2}^{2}}) \end{array}$$

**Figure 3:**
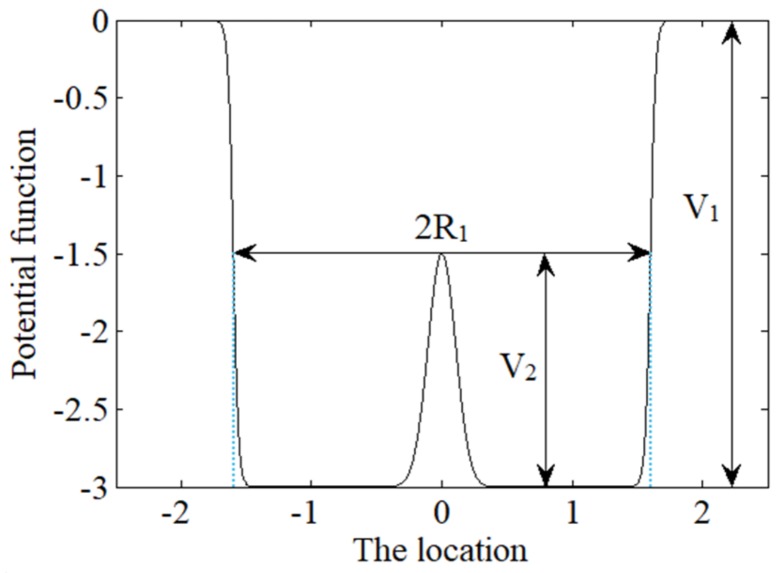
The shape of Woods-Saxon and Gaussian (WSG) potential function.

The shape of the new potential function is shown in Figure 3, where the corresponding parameters are $V_{1}=3$, $R_{1}=1.6$, a=0.02, $V_{2}=1.5$, $R_{2}=0.15$. It can be seen from the figure below that it is a bistable potential well model with two symmetrical potential wells and $V_{2}$ is the height of the potential barrier. The width of each potential well and the distance between the two potential wells can be changed by adjusting $R_{1}$ and $R_{2}$. So, the shape of the potential well model is controlled by five parameters and they are not coupled, which means the shape of the potential well can be adjusted by changing any single parameter. As the new bistable potential well model is formed by adding GP into the WSP model, so it not only has the advantage of the Woods-Saxon single potential well but also has the advantage of the traditional bistable potential. Obviously, it is more likely to achieve stochastic resonance state if the well wall is steeper and the bottom is flatter. Therefore, the weak periodic signal can be well processed and enhanced by stochastic resonance with this kind of potential well.

### 3.4. Stochastic Resonance System

**Figure 4:**
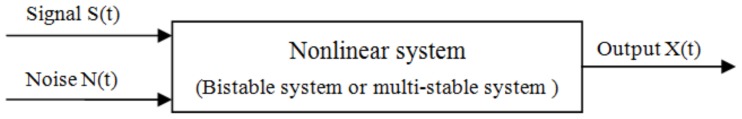
The general structure block diagram of stochastic resonance.

As the bistable stochastic resonance can enhance the weak low frequency signal by utilizing the noise energy reasonably, it can be thought as a nonlinear low pass filter in some literature [15,23]. Therefore, according to the careful study of some model based research works on the signal processing and analysis [24,25,26], the bistable stochastic resonance here is thought as a nonlinear system and the noisy signal [27] containing the periodic signal and strong noise is considered as the input of this nonlinear system shown in Figure 4. Then, the weak characteristic frequency can be obviously enhanced in the output signal. Since there will be multiple stable points for the high order potential function, the nonlinear system also includes multi-stable system besides the bistable system. 

Taking Brown particle’s over damped motion excited by noise and external driving force in a bistable system into consideration, the stochastic resonance system can be described by the following equation:

(10) $$\frac{dx}{dt}=-V^{\prime }(x)+A_{0}\sin(2\pi f_{0}t+\varphi )+n(t)$$

Wherein V(x) is the nonlinear bistable potential function and the traditional V(x) is shown by Equation (11).

(11) $$\begin{array}{cc} V(x)=-\frac{a}{2}x^{2}+\frac{b}{4}x^{4} & a>0,\;b>0 \end{array}$$

$n(t)=\sqrt{2D}\xi (t)$, E[n(t)n(t+τ)]=2Dδ(τ) in Equation (10), where D represents the noise intensity, ξ(t) is white noise with zero mean and unit variance. Parameters a and b are real and positive structural coefficients. $A_{0}(t)$ is the amplitude of periodic signal and $f_{0}$ is the driving frequency. Substituting Equation (11) into Equation (10) and the following Equation (12) can be obtained.

(12) $$\frac{dx}{dt}=ax-bx^{3}+A_{0}\sin(2\pi f_{0}t+\varphi )+\sqrt{2D}\xi (t)$$

Equation (12) above represents a classical bistable stochastic resonance system through the nonlinear Langevin equation. If WSG bistable potential well model is used to replace the traditional potential function V(x), then the new system can be called WSG stochastic resonance system. Accordingly, the equation can be expressed as follows.

(13) $$\begin{array}{cc} \frac{dx}{dt}= & -\frac{V_{1}}{a}\mathrm{sgn}(x)\exp\left(\frac{\left|x\right|-R_{1}}{a}\right){\left(1+\exp\left(\frac{\left|x\right|-R_{1}}{a}\right)\right)}^{-2}\\ & +\frac{2V_{2}x}{R_{2}^{2}}\exp\left(-\frac{x^{2}}{R_{2}^{2}}\right)+A_{0}\sin(2\pi f_{0}t+\varphi )+n(t) \end{array}$$

Wherein, sgn(x) is the symbolic equation and $\mathrm{sgn}(x)=\left\{ \begin{array}{ll} 1 & x>0\\ 0 & x=0\\ -1 & x<0 \end{array}\right.$.

It can be seen from Equation (13) that the system output depends on the potential model and input signals. The difference between WSG stochastic resonance system and the classical system lies in the potential model. In fact, the occurrence of stochastic resonance is determined by the combined effect of the system, input signal and the noise. The stochastic resonance phenomenon can be described by the oscillation of Brown particles under a variety of excitation (including potential well force, periodic force and noise force) in a created special scene. In all forces, potential well force is obtained from the first-order derivative of potential function. In reality, the periodic force and the noise power are fixed, so the effectiveness of the stochastic resonance system is largely determined by the potential well force.

In general, if the potential well is too wide, the particle can’t reach the potential well wall of the other end or the barrier. It also means the particle needs greater resilience to help its periodic motion in a cycle. On the other hand, if the well is too narrow, the particle can’t reach the expected position along the right path in a cycle, because the wall of the potential well will force the particle return back to its original position in advance. Similarly, the excessively high barrier will make it difficult for the particles to move back and forth in two potential wells, as a result they will only do the reciprocating motion in one potential well which will have no effect on enhancing the weak signal from strong noise. If the barrier is too low, the motion of particles will have no periodicity and just be controlled by the effect of noise. That is to say, the output signal is disordered. In addition, if the potential well wall is too steep, the produced great resilience will further lead to the rebound of the particles. Conversely, it will not provide sufficient energy for particles to complete the periodic movement if the well wall is very flat. In conclusion, the periodic motion of the particles and the amplification of a weak characteristic signal can be ensured only when the shape of the potential model reaches the best state. Therefore, the corresponding parameter optimization method is necessary.

### 3.5. Weak Characteristic Extraction Based on CWT-WSG Stochastic Resonance Method and GA

**Figure 5:**
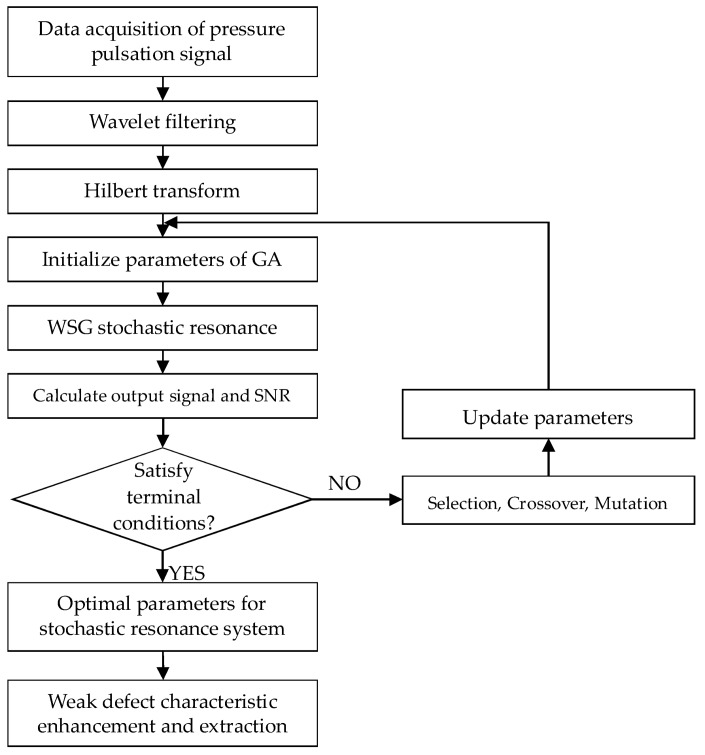
The flow chart of WSG stochastic resonance method based on genetic algorithm (GA).

Based on the CWT filtering and WSG stochastic resonance feature enhancement method, the CWT-WSG method is proposed to extract the weak characteristic information. As the output signal quality of WSG stochastic resonance method is determined by potential parameters, the optimal output signal can be obtained by tuning related potential parameters. Therefore, Genetic algorithm (GA) is studied here for the determination of optimal parameters. Signal-to-noise ratio (SNR) of the output signal shown by Equation (14) is set as the objective function in GA.

(14) $$SNR=10\log_{10}\left(\frac{A_{c}}{\sum_{i=1}^{N/2}A_{i}-A_{c}}\right)$$

where $A_{c}$ is the amplitude of weak characteristic frequency in the frequency spectrum. N represents the length of signal and $A_{i}$ stands for the amplitude of each spectrum line in the frequency spectrum of output signal. The purpose of GA is to search for optimal parameters so that the optimal SNR can be obtained, as the larger SNR means the better characteristic enhancement performance. The flowchart of the proposed method is illustrated in Figure 5 and detailed procedures are described as follows.

(1) Signal pre-processing based on CWT: As blade passing frequency, a high-frequency component calculated by multiplying blade numbers with rotating frequency, is the main frequency during the operation of the centrifugal compressor, the low-frequency component caused by blade defect may be modulated to the blade passing frequency [5,25]. In addition, the information produced by blade crack is usually very weak and overwhelmed by strong noise. Therefore, CWT is used to filter the acquired pressure pulsation signal first and the scales around blade passing frequency are selected from the calculated scalogram to reconstruct the processed signal. Then Hilbert transform is used for the reconstructed signal to obtain the envelope signal.
(2) Selection of parameters to be optimized: As $V_{1},V_{2},R_{1},R_{2},a$ can all affect the result, they are selected as the parameters which need to be optimized by GA. To meet the requirement of small parameters (including low driving frequency, low amplitude and low noise intensity) of stochastic resonance, frequency shifted and rescaling transform is needed. As the change of rescaling factor k can also affect the output performance, therefore, k is also selected to be the parameter to be optimized.
(3) Parameters setting and initialization for GA algorithm: Several required parameters need to be set when using GA algorithm. They are population size N, number of variables $N_{\mathrm{var}}$, length of binary encoding L, the generation gap rate between offspring and fathers $G_{\mathrm{gap}}$, the maximum number of generations $G_{\max}$ and the ranges of $V_{1},V_{2},R_{1},R_{2},a,k$. Here, set N=200, $N_{\mathrm{var}}=6$, L=25, $G_{\mathrm{gap}}=0.9$, $G_{\max}=50$ respectively for the high convergence speed and accuracy of GA algorithm. The ranges of $V_{1},V_{2},R_{1},R_{2},a$ are all initialized as [0,10] to ensure the occurrence of various shapes of potential functions and the range of rescaling factor k is set as [0,1000] to rescale the characteristic frequency to be small parameter (f≪1 Hz).
(4) Output signal calculation and parameters update: Each produced chromosome is brought into Equation (13) and then this equation is solved by using the Fourth-order Runge-Kutta method to obtain the output signal, where the processed envelope signal is set as the input weak periodic signal. Next, calculate the SNR of output signal with Equation (14) and set it as objective function value. Searching the optimal parameters based on fitness function and updating the generation. Repeat the above procedures until the optimal parameters are obtained.
(5) Weak characteristic enhancement and extraction: Based on the obtained optimal potential parameters $V_{1},V_{2},R_{1},R_{2},a$ and rescaling factor k, take the filtered envelope signal into Equation (13) and calculate the output signal. Then, conducting Fourier transform on the output signal, as a result, the weak characteristic frequency can be enhanced and extracted.

### 3.6. The Blade Weak Defect Identification Based on the Proposed Method

**Figure 6:**
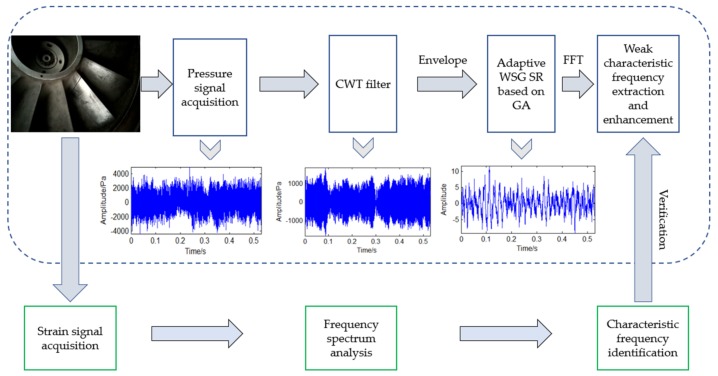
The system diagram on the application and evaluation for the proposed method.

As the weak characteristic frequency induced by the blade crack defect is usually difficult to be identified, the proposed method is used here to improve this problem. Considering the strain method is not suitable for the long-term condition monitoring of all the blades, pressure pulsation signal is adopted in this paper. Then, the proposed CWT-WSG SR method combined with GA is used to enhance and extract the weak defect characteristic frequency from the pressure signal. Strain data will also be sampled and analysed to evaluate the reliability and effectiveness of the analysis result obtained by using the proposed method. In the condition that the consistent defect characteristic frequency can be extracted from both strain signal and pressure pulsation signal with the proposed method, it can be verified this method is effective and has potential in the application of long-term condition monitoring and weak defect warning for large-scale centrifugal compressor blades. The detailed scheme is shown by the following system diagram seen as Figure 6.

## 4. Simulation Signal Analysis

In order to illustrate the effectiveness of the proposed CWT-WSG stochastic resonance method, a simulation signal is analysed first. Considering the actual situation that amplitude modulation phenomenon will occur if there is defect in the blade, an amplitude-modulated signal is constructed shown by Equation (15).

(15) $$s(t)=A(1+B\cos(2\pi f_{e}t))\sin(2\pi f_{c}t).$$

**Figure 7:**
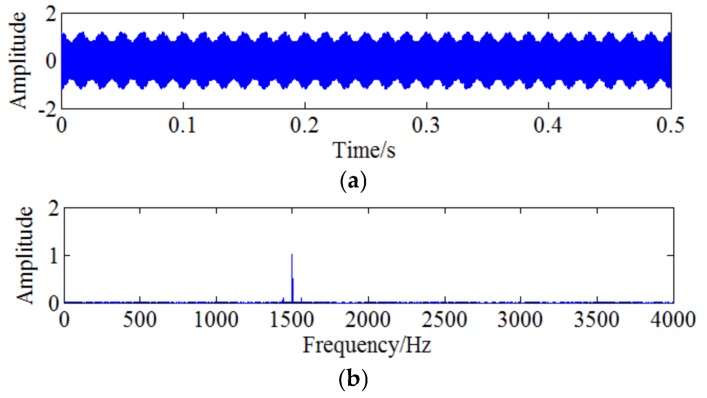

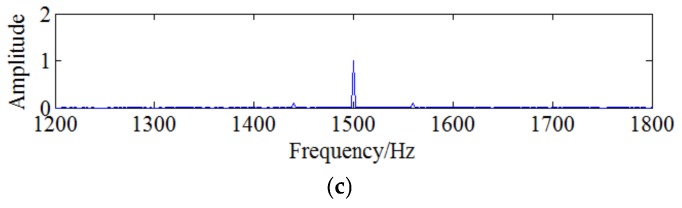
Simulation signal without noise: (**a**) Amplitude modulated signal without noise; (**b**) The frequency domain of simulation signal; (**c**) The larger image of frequency domain.

**Figure 8:**
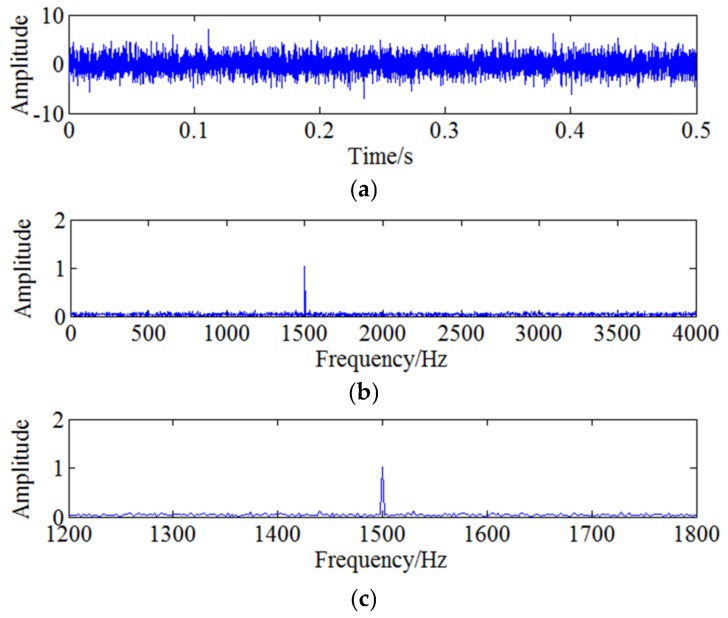
Simulation signal with noise: (**a**) The time domain signal with noise; (**b**) The frequency domain; (**c**) The larger image of frequency domain.

Wherein the carrier frequency $f_{c}=1500$ Hz and the modulation frequency $f_{e}=60$ Hz. Parameters A=1 and B=0.2. The sampling frequency is $f_{s}=10240$ Hz and the number of sampling points is 5120. The obtained amplitude-modulated signal is shown as Figure 7a and it is analysed by FFT to obtain the frequency spectrum which is shown as Figure 7b, where 1500 Hz is the main frequency and 60 Hz is the modulation frequency. Amplifying the frequency information around 1500 Hz to obtain Figure 7c and it can be clearly seen that there exists sideband around 1500 Hz, which indicates 60 Hz is modulated to the carrier frequency. In order to simulate the actual situation, strong Gaussian white noise with SNR = −9 dB is added to the simulation signal and the obtained time domain signal is shown as Figure 8a. Figure 8b shows the corresponding frequency domain. It can be found that the side band information is difficult to be identified due to the noise interference. Amplifying the frequency domain near 1500 Hz shown by Figure 8c, however, the characteristic frequency is still difficult to obtain through side-band analysis due to the strong noise interference.

**Figure 9:**
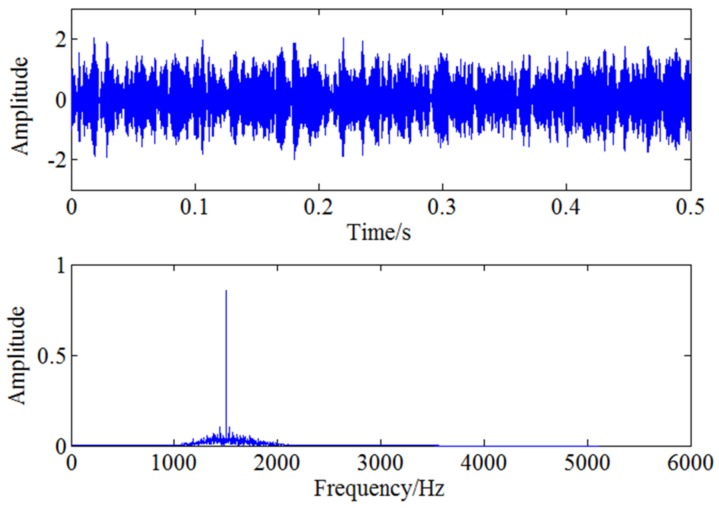
The time and frequency domain of signal after continuous wavelet transform (CWT).

**Figure 10:**
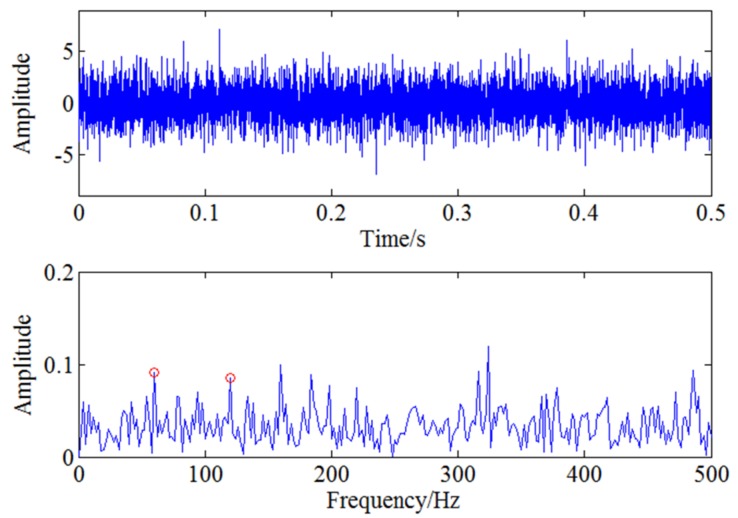
The time and frequency domain after CWT-envelope analysis.

In order to extract the weak modulation frequency 60 Hz, CWT method is used to filter the signal around 1500 Hz first shown in Figure 9. Then envelope spectrum analysis is used for the signal and the result is shown as Figure 10. It can be seen from Figure 10, although the characteristic frequency 60 Hz and its harmonic frequency 120 Hz marked with red circles can be extracted. However, due to the noise interference, the useful feature information is not clear and submerged in strong noise. Therefore, the characteristic frequency needs to be analysed further with feature enhancement algorithm.

**Figure 11:**
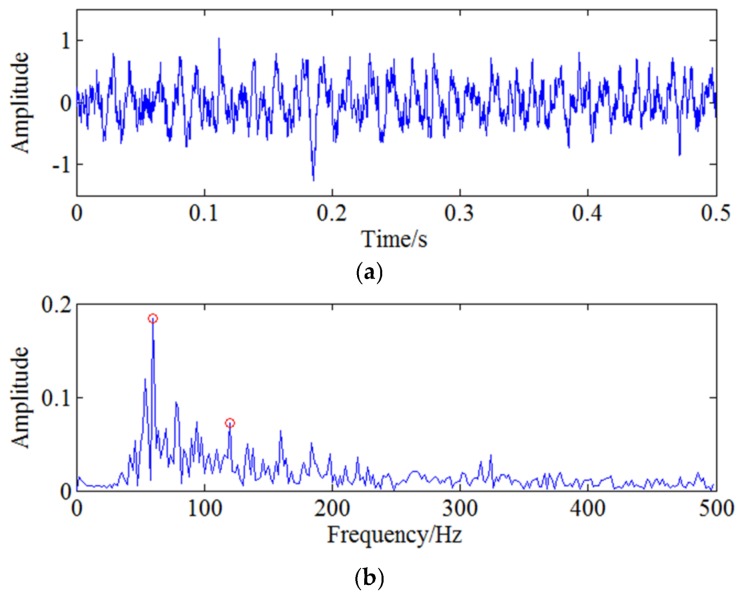
The processed signal with stochastic resonance: (**a**) The output signal of stochastic resonance system; (**b**) The frequency domain of output signal.

For further extracting the characteristic frequency, WSG stochastic resonance model is constructed to process the envelope signal. Related optimal parameters of the WSG model are set as $V_{1}=3.9,\;V_{2}=0.6,\;R_{1}=8.8,\;R_{2}=9.7,\;a=1.4$ based on GA algorithm and the rescaling factor k is set as 660. Calculate Equation (13) with the determined parameters and then the output signal shown in Figure 11a can be obtained through this nonlinear system. Spectrum analysis is used for the output signal to obtain the result shown in Figure 11b. It can be clearly seen from the figure that the motion of the particle in two potentials reaches resonance state well, so the characteristic frequency 60 Hz is enhanced obviously and easier to be identified after WSG stochastic resonance system. Therefore, the proposed CWT-WSG stochastic resonance method is verified effective and feasible to enhance the weak characteristic frequency. Based on the simulation analysis, it can be proved the CWT-WSG stochastic resonance method is helpful for weak feature enhancement and detection. Moreover, the result also shows the proposed method can provide a new idea for incipient weak defect detection of the centrifugal compressor blade. 

## 5. Application for the Actual Compressor Signal

### 5.1. The Experiment and Data Acquisition System

**Figure 12:**
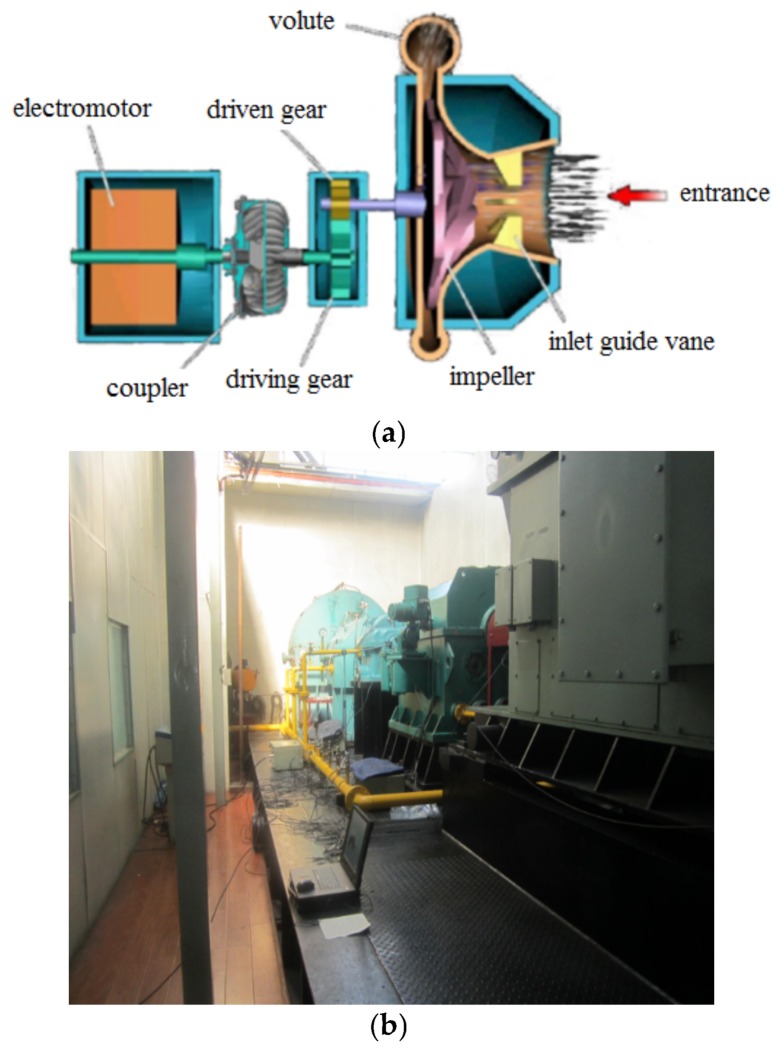
The centrifugal compressor test rig: (**a**) Schematic for the test-rig; (**b**) Experimental centrifugal compressor.

**Figure 13:**
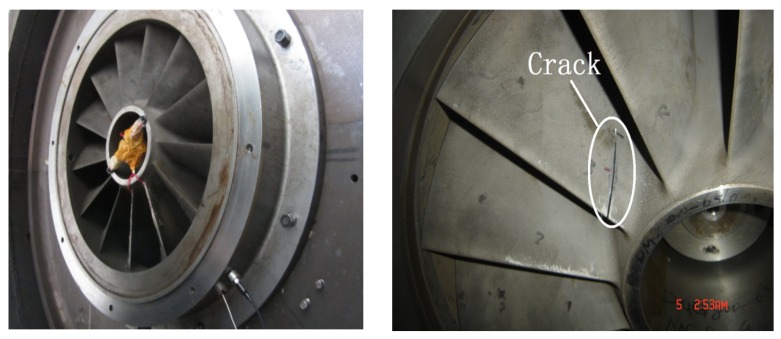
The impeller and crack.

The centrifugal compressor test rig is shown in Figure 12 and it can be seen from the figure that the test rig consists of motor, coupler, gear box, impeller and so forth. The impeller is a semi-open one with 13 blades and 800 mm diameter. It is an experimental impeller used for engineering study. The impeller is driven by the motor and its speed can be adjusted through the coupler and gear box with the speed range from 500 rpm to 9000 rpm. In this experiment, the rotating speed of the impeller is set as 5000 rpm and the corresponding shaft frequency and blade passing frequency are 5000/60=83.3 Hz and 5000×13/60=1083.3 Hz respectively. The detailed experimental parameters are given in Table 1. In order to simulate the blade crack defect, a notch is machined on one blade of the normal semi-open impeller. Strain signal of the blade and pressure pulsation signal of the airflow are both collected during the rotation of the impeller and the relationship between the crack and the acquired signal is studied with feature extraction method so that the crack can be accurately identified at initially. The length of the crack shown in Figure 13 is 70 mm. As the thickness of the blade is very large, the abnormal vibration amplitude caused by the blade crack is actually very weak, what’s worse, the useful defect characteristic information contained indirectly in pressure pulsation signal will be much weaker. Moreover, there is strong noise interference during the high-speed rotation of the impeller. Therefore, the defect characteristic frequency is difficult to identify with traditional signal processing methods. In order to improve this problem, the proposed CWT-WSG stochastic resonance method will be applied on the acquired pressure pulsation signal.

**Figure 14:**
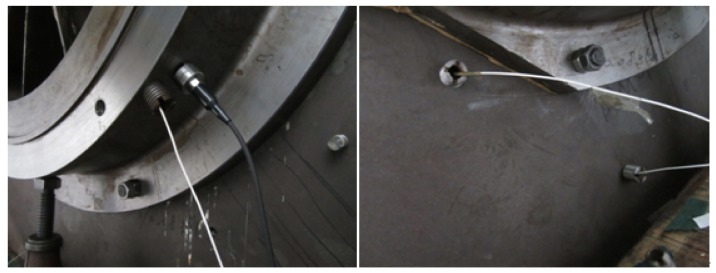
The installation location of sensors.

In order to accurately collect crack defect information of the blade, appropriate data acquisition system and sensors are needed to collect and read data. NI4472 eight channel dynamic signal acquisition module, a synchronous signal acquisition module with 110 dB dynamic range, 24-bit resolution and 102.4 kHz sampling frequency, is selected as the hardware and it constitutes the test system with the DASP software of East China Institute of Noise and Vibration during the experiment. Dynamic pressure sensors are used to monitor the pressure pulsation signal in the experiment. The principle of the sensor is that sound wave can cause the vibration of air. The pulsant air will then press the diaphragm and the diaphragm will generate an electric signal under the action of the mechanical force. The test results are closely related to the arrangement of measuring points. How to arrange the data points is a key factor to the success of the test and it is also an important guarantee for the validity and accuracy of the data. In addition, measuring points positions play an important role for the effective extraction of the blade crack information in the subsequent data processing step. Therefore, it is necessary to design measuring points reasonably before the experiment. The reasonable positions of the measuring points must meet the following requirements: (1) The layout of measuring points need to meet the requirements of the test; (2) The positions of measuring points need to ensure the authenticity and validity of the test data; (3) The positions also need to meet the requirements of site conditions, which means the test equipment should be placed on the points easily and safely. The pressure sensors are placed on the impeller inlet, the diffuser inlet and exit respectively to monitor the pressure pulsation signal in this experiment and Figure 14 shows the installation positions of pressure sensors.

Considering the defect characteristic is weak, the used pressure sensors should be placed near the position of the rotating impeller. According to the actual situation of the Φ800 test rig, radial holes are machined in the selected measuring points so that pressure sensors can be embedded. The probe of sound pressure transducer stretches a certain distance into the pipe inner wall, which is very close to the impeller so that the useful pressure signal containing the crack information can be obtained. PCB 106B is chosen as the sound pressure sensor in the experiment. The maximum dynamic pressure it can withstand is 68.9 KPa.

### 5.2. Pressure Pulsation Signal Analysis

**Figure 15:**
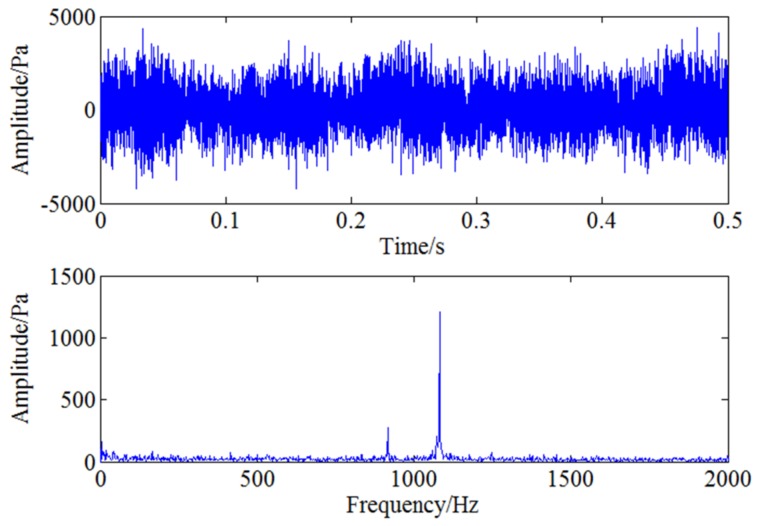
Time and frequency domain of pressure pulsation signal under 5000 rpm.

Pressure pulsation signal is generated due to the interaction between the rotor and the stator inside the compressor and the main characteristic frequencies in normal operation conditions are blade passing frequency and its harmonic frequencies. In order to get crack information of the cracked blade comprehensively, a test is carried out on a compressor with a cracked blade at 5000 rpm working condition. Pressure pulsation signal of the three positions mentioned above are collected synchronously with multi-channel acquisition system. Figure 15 shows the time and frequency domain of the sampled pressure pulsation signal at 5000 rpm. As the blade passing frequency 1083.3 Hz belongs to high frequency component and the characteristic frequency induced by blade crack is a low frequency component, so this characteristic frequency will be modulated to blade passing frequency during the rotation of the blade. However, the obvious modulated frequency in Figure 15 is two times shaft frequency 166.6 Hz which may be caused by the misalignment of the shaft and there aren’t other obvious modulated frequencies except it, which means the weak feature information caused by blade crack can’t be identified by general spectrum analysis.

**Figure 16:**
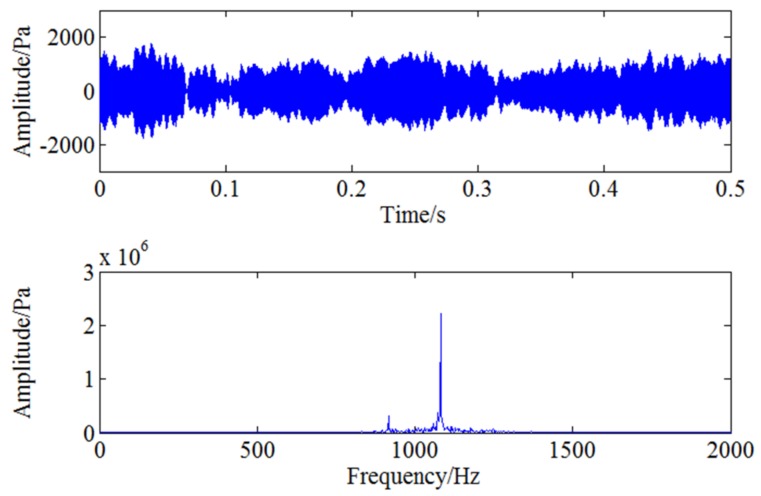
The filtered signal through CWT.

**Figure 17:**
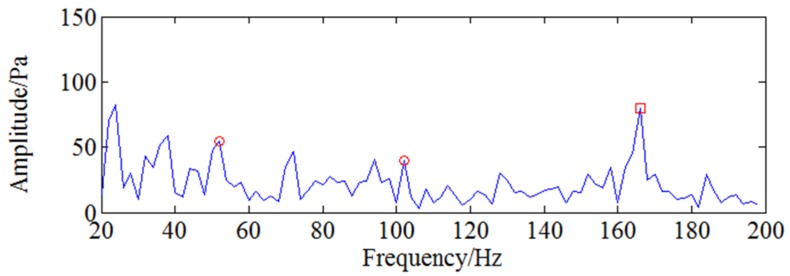
Envelope analysis for filtered pressure pulsation signal under 5000 rpm.

Therefore, the feature frequency caused by blade crack needs to be demodulated with further analysis. As the blade passing frequency is 1083.33 Hz, CWT is first used to filter the signal around 1083 Hz and the filtered signal is reconstructed through continuous wavelet inverse transform shown as Figure 16. Envelope spectrum analysis is then used for the filtered signal and the result is shown in Figure 17. The two frequencies marked with red circles in Figure 17 are 53 Hz and 106 Hz respectively, that is to say, there exists typical frequency multiplication relationship between the fundamental frequency 53 Hz and the harmonic frequency 106 Hz. It can be determined that 53 Hz is the characteristic frequency excited by blade crack which will be verified by strain test shown in the following but it is not clear enough due to strong noise interference. Moreover, it can also be found the two times shaft frequency component 166.6 Hz marked with red rectangle is more obvious compared with 53 Hz. Therefore, WSG stochastic resonance model is absolutely necessary to further process the envelope signal for the purpose of enhancing the weak characteristic frequency 53 Hz.

**Figure 18:**
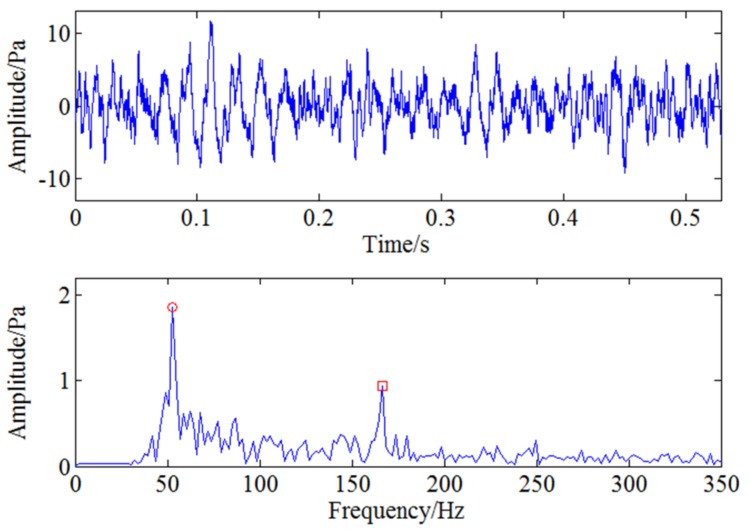
The output signal of stochastic resonance model under 5000 rpm.

According to the optimization result of GA, the optimal parameters of the WSG stochastic resonance model are set as $V_{1}=0.7,V_{2}=3.1,R_{1}=6.7,R_{2}=8.9,a=0.14$ respectively and the corresponding rescaling factor k is set as 1000 to change 53 Hz into small parameter. Calculate the output signal and go ahead with spectrum analysis by Fourier transform. The obtained output signal and the corresponding spectrum are shown by Figure 18 and it can be seen the characteristic frequency 53 Hz marked by red circle is more obvious than two times shaft frequency 166.6 Hz marked by red rectangle. Comparing Figure 17 and Figure 18, it can be clearly found the characteristic frequency is much easier to be identified with the further processing of stochastic resonance. Therefore, it can be known the effective weak crack information enhancement is achieved by the proposed CWT-WSG stochastic resonance method.

It can be concluded from the above analysis that a low vibration frequency 53 Hz is generated for the cracked blade during the rotation of the impeller. This low frequency vibration of the cracked blade will act on the air and can be found from pressure pulsation signal nearby. As blade passing frequency belongs to high frequency component, the 53 Hz will be modulated to it seen as the sideband. CWT-envelope method can’t extract this weak defect frequency effectively because of the strong noise interference. Conversely, this characteristic frequency can be found easily with further processing by CWT-WSG stochastic resonance method. In order to further verify the validity of the result, strain test and analysis are also conducted described as follows.

### 5.3. Verification Analysis of Strain Data

**Figure 19:**
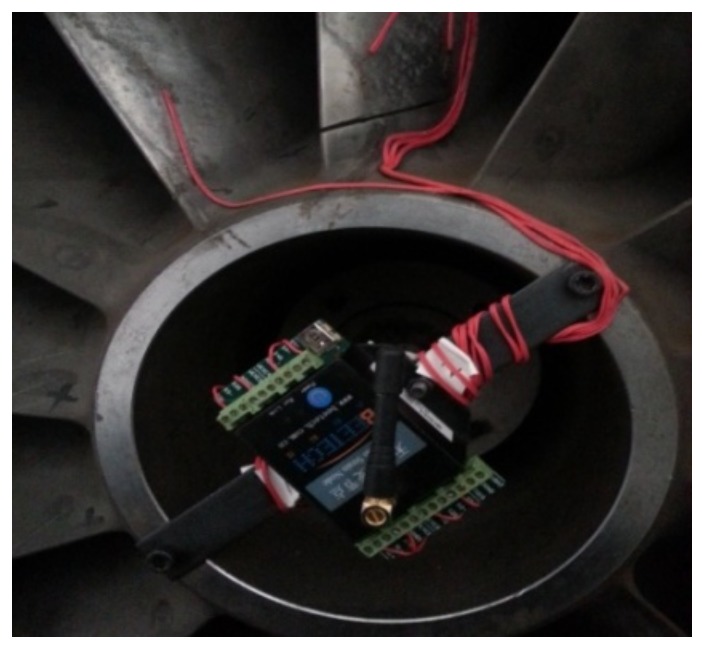
The installation of wireless strain node.

**Figure 20:**
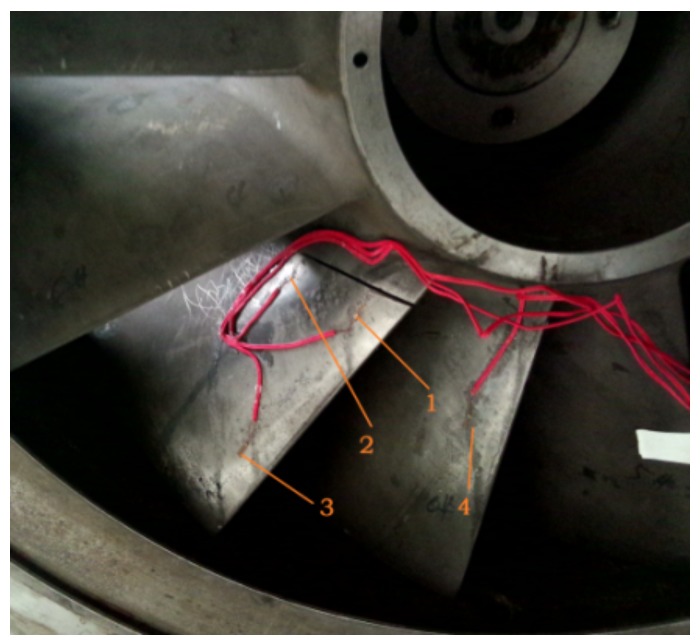
The installation position of strain gauges.

The blade’s strain test acquisition system consists of a SG403/SG404 four-channel wireless strain sensor node, a wireless receiving gateway, BeeDate wireless sensor network software and BF120-2AAGN20-W strain gauges. The installation of the wireless strain node is shown in Figure 19. Strain signal is sampled both from the cracked blade and the adjacent normal blade and the installation positions of strain gauges are shown in Figure 20, wherein position 1,2,3 are attached on the cracked blade and position 4 is on a normal blade.

**Figure 21:**
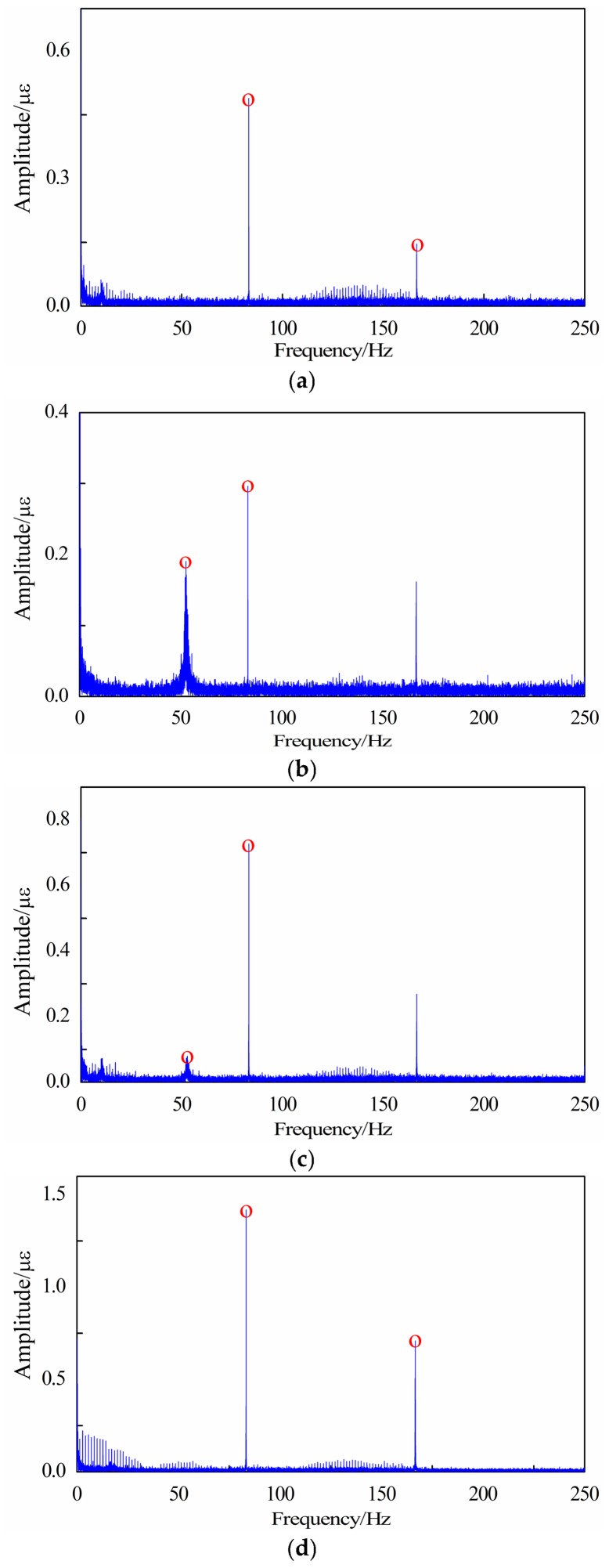
The experimental strain results under 5000 rpm: (**a**) The experimental strain result of position 1; (**b**) The experimental strain result of position 2; (**c**) The experimental strain result of position 3; (**d**) The experimental strain result of position 4.

Analysing the strain signal obtained at 5000 rpm working condition. The acquired spectrums are shown in Figure 21 and it can be seen from the result that the blade’s defect frequency of 53 Hz can be detected at position 2 and position 3. This characteristic frequency at measuring point 2 which is near the crack is more obvious but it cannot be found at point 4, which is on the normal blade. Based on the analysis result of the strain signal, it can be found that the low frequency of 53 Hz exists both in the strain signal and the pressure pulsation signal. In order to further ensure the reliability of the identified defect frequency, the strain signal is also sampled and analysed at 4000 rpm and 4500 rpm working conditions. The defect characteristic frequency can still be obviously extracted from the frequency spectrums. Moreover, there will exist a low frequency component modulated to the high frequency component-blade passing frequency in the condition that the impeller blade is cracked according to References [5,25]. Therefore, it can be proved that the proposed method is reliable and effective for the condition monitoring and weak defect identification of the incipient blade crack.

## 6. Conclusions

Condition monitoring and incipient weak defect warning for centrifugal compressor blades is always a big challenge as the impeller works in a closed space, which means the signal that can effectively reflect the blade’s state is difficult to acquire. Traditional non-destructive testing and evaluation (NDT&E) methods can determine the blade defect effectively, however, these methods can only be performed during the shutdown period of the compressors, which are time-consuming and costly. Therefore, they are not suitable for the long-term on-line condition monitoring of blades. Considering the interaction between the rotating impeller and the airflow nearby, the blade’s abnormal vibration induced by the crack defect will affect the around pressure pulsation of the airflow. Therefore, pressure pulsation signal is used for the on-line condition monitoring and defect characteristic extraction in this paper. However, as the characteristic information is usually weak together with strong noise, traditional signal processing methods cannot identify it effectively. CWT is used to pre-process the sampled signal and WSG stochastic resonance is then adopted to enhance the weak characteristic frequency. GA is used to obtain the optimal parameters for improving the enhancement performance of this stochastic resonance system. The accuracy of the proposed method is verified by the strain experiment. In conclusion, this paper provides a new idea for the weak defect characteristic enhancement and the identification of centrifugal compressor blades. For future research, a study on multi-cracks of different sizes should be conducted by using a pressure pulsation signal, which can make this method more helpful in practice. In addition, as the synergistic effect of noise can better improve the characteristic enhancement performance of the stochastic resonance system, multi-scale noise tuning can also be further studied in the future to achieve a better blade defect detection performance for the proposed CWT-WSG stochastic resonance method. 

## Figures and Tables

**Table 1:** Test parameters.

Speed	Blade Number	Shaft Frequency	Blade Passing Frequency	The Length of Crack
5000 rpm	13	83.3 Hz	1083.3 Hz	70 mm

## References

[1] Eisinger F.L. (2002). Acoustic Fatigue of Impellers of Rotating Machinery. J. Press. Vessel Technol..

[2] Witek L. (2009). Experimental Crack Propagation and Failure Analysis of the First Stage Compressor Blade Subjected to Vibration. Eng. Fail. Anal..

[3] Li Z., Jiang Y., Guo Q., Hu C., Peng Z. (2018). Multi-Dimensional Variational Mode Decomposition for Bearing-Crack Detection in Wind Turbines with Large Driving-Speed Variations. Renew. Energy.

[4] Saravanan K., Sekhar A. (2013). Crack Detection in a Rotor by Operational Deflection Shape and Kurtosis Using Laser Vibrometer Measurements. J. Vib. Control.

[5] Rao A.R., Dutta B. (2012). Vibration Analysis for Detecting Failure of Compressor Blade. Eng. Fail. Anal..

[6] Egusquiza E., Valero C., Huang X., Jou E., Guardo A., Rodriguez C. (2012). Failure Investigation of a Large Pump-turbine Runner. Eng. Fail. Anal..

[7] Joosse P.A., Blanch M.J., Dutton A.G., Kouroussis D.A., Philippidis T.P., Vionis P.S. (2002). Acoustic Emission Monitoring of Small Wind Turbine Blades. J. Sol. Energy Eng..

[8] Pawar P.M., Jung S.N. (2008). Support Vector Machine Based Online Composite Helicopter Rotor Blade Damage Detection System. J. Intell. Mater. Syst. Struct..

[9] König S., Petry N., Wagner N.G. Aeroacoustic Phenomena in High-pressure Centrifugal Compressors—A Possible Root Cause for Impeller Failures. Proceedings of the 38th Turbomachinery Symposium.

[10] Petry N., Benra F.K., König S. Experimental Study of Acoustic Resonances in the Side Cavities of a High-pressure Centrifugal Compressor Excited by Rotor/stator Interaction. Proceedings of the ASME Turbo Expo 2010: Power for Land, Sea, and Air.

[11] Leng Y.G., Leng Y.S., Wang T.Y., Guo Y. (2006). Numerical Analysis and Engineering Application of Large Parameter Stochastic resonance. J. Sound Vib..

[12] Benzi R., Sutera A., Vulpiani A. (1981). The Mechanism of Stochastic Resonance. J. Phys. A Math. Gen..

[13] He Q., Wang J., Liu Y., Dai D., Kong F. (2012). Multiscale Noise Tuning of Stochastic Resonance for Enhanced Fault Diagnosis in Rotating Machines. Mech. Syst. Signal Process..

[14] Qiao Z., Lei Y., Lin J., Jia F. (2017). An adaptive unsaturated bistable stochastic resonance method and its application in mechanical fault diagnosis. Mech. Syst. Signal Process..

[15] Lei Y., Qiao Z., Xu X., Lin J., Niu S. (2017). An Underdamped Stochastic Resonance Method with Stable-state Matching for Incipient Fault Diagnosis of Rolling Element Bearings. Mech. Syst. Signal Process..

[16] Tan J., Chen X., Wang J., Chen H., Cao H., Zi Y., He Z. (2009). Study of Frequency-shifted and Re-scaling Stochastic Resonance and Its Application to Fault Diagnosis. Mech. Syst. Signal Process..

[17] Zhang H., He Q., Lu S., Kong F. (2014). Stochastic Resonance with a Joint Woods-Saxon and Gaussian Potential for Bearing Fault Diagnosis. Math. Probl. Eng..

[18] Zhang X., Hu N., Hu L., Cheng Z. (2013). Multi-scale Bistable Stochastic Resonance Array: A Novel Weak Signal Detection Method and Application in Machine Fault Diagnosis. Sci. China Technol. Sci..

[19] He Q., Wang J. (2012). Effects of Multiscale Noise Tuning on Stochastic Resonance for Weak Signal Detection. Digit. Signal Process..

[20] Qiao Z., Lei Y., Lin J., Niu S. (2016). Stochastic Resonance Subject to Multiplicative and Additive Noise: The Influence of Potential Asymmetries. Phys. Rev. E.

[21] Qin Y., Zhang Q., Mao Y., Tang B. (2016). Vibration Component Separation by Iteratively Using Stochastic Resonance with Different Frequency-scale Rations. Measurement.

[22] Hu B., Li B. (2015). Blade Crack Detection of Centrifugal Fan Using Adaptive Stochastic Resonance. Shock Vib..

[23] Lu S., He Q., Kong F. (2015). Effects of Underdamped Step-varying Second-order Stochastic Resonance for Weak Signal Detection. Digit. Signal Process..

[24] Fan M., Cao B., Yang P., Li W., Tian G. (2015). Elimination of Liftoff Effect Using a Model-based Method for Eddy Current Characterization of a Plate. NDT E Int..

[25] Fan M., Wang Q., Cao B., Ye B., Sunny A.I., Tian G. (2016). Frequency Optimization for Enhancement of Surface Defect Classification Using the Eddy Current Technique. Sensors.

[26] Yu J., Chang J., Su B. (2017). Modeling of whole-space transient electromagnetic responses based on FDTD and its application in the mining industry. IEEE Trans. Ind. Inf..

[27] Malekian R., Bogatinoska D.C., Karadimce A., Ye N., Trengoska J., Nyako W.A. (2015). A Novel Smart ECO Model for Energy Consumption Optimization. Elektronika ir Elektrotechnika.

